# Animacy cues facilitate 10-month-olds' categorization of novel objects with similar insides

**DOI:** 10.1371/journal.pone.0207800

**Published:** 2018-11-26

**Authors:** Nina Anderson, Kristinn Meagher, Andrea Welder, Susan A. Graham

**Affiliations:** 1 Department of Psychology, University of Calgary, Calgary, Alberta, Canada; 2 Owerko Centre, Alberta Childrens’ Hospital Research Institute, University of Calgary, Calgary, Alberta, Canada; SWPS University of Social Sciences and Humanities, POLAND

## Abstract

In this experiment, we examined whether sensitivity to the relevance of object insides for the categorization of animate objects is in place around 10 months of age. Using an object examining paradigm, 10-month-old infants’ (N = 58) were familiarized to novel objects with varying outward appearances but shared insides in one of three groups: No cues, Eyes, and Cue control. During test trials, infants were presented with a novel in-category test object followed by an out-of-category test object. When objects were presented with animacy cues (i.e., Eyes), infants categorized the objects together. In contrast, when objects were presented without any added cues or when they were presented with a shared perceptual marker (Cue control, i.e., plastic spoons placed on top of the objects), infants showed no evidence of categorization. These results indicate that by 10 months of age, eyes signal to infants that objects share some kind of uniting commonality that may not be obvious or readily perceptually available.

## Introduction

Well before they reach kindergarten, children have a well-developed understanding of the distinction between animates and inanimates (for reviews see [1, 2]). Research has shown that an appreciation of this distinction can guide children’s reasoning about objects and object categories [3–10]. For example, toddlers will extend novel words to artifacts based on shared shape but will extend novel words to animates based on both shape and texture, indicating that they shift their basis for naming according to the conceptual status of the object [11]. In this experiment, we examine the question of how animacy guides object categorization, focusing specifically on whether animacy cues lead 10-month-olds to categorize objects based on similar insides.

As noted above, young children reason differently about animates and inanimates. During the preschool years, children privilege internal features when determining an object’s identity when they are considering animate objects, but not inanimate objects [12]. Similarly, preschoolers exhibit a tendency to rely on internal information when determining category membership for animate beings [13–15]. For example, Diesendruck et al. [16] demonstrated that when 3- to 5-year-olds learnt about the internal similarities between two animals (e.g., both had bones and blood), they were more likely to judge that these animals belonged to the same category compared to preschoolers who heard a superficial similarity described (e.g., both lived in a cage). In contrast, the kind of property (internal versus superficial) shared by two pictured artifacts did not influence categorization (see also [17]). Furthermore, 5-year-olds will use internal properties to categorize animals, but not artifacts, when internal and external properties are in competition [18].

The research described above demonstrates that preschoolers appreciate the importance of internal features for animates. A number of studies have demonstrated that this understanding emerges during infancy (see [19] for a review). For example, 8-month-olds expect self-propelled agents to possess insides, and exhibit surprise when an item exhibiting self-propulsion and agency is shown to be hollow [20].When the item has only one of these two properties (i.e., the item is self-propelled but lacks agency, or exhibits agency but not self-propulsion), infants do not appear to hold expectations regarding the item’s insides. However, when the self-propelled item is covered with fur, 8-month-olds expect the object to have insides, thus suggesting that the fur is used as an animacy cue. Similarly, 14-month-olds associate self-propelled motion with the presence of an internal feature of an object, and attend more towards internal features when an object exhibits self-generated behaviors [21]. In addition to expecting animate beings to possess insides, infants will use the insides to determine an animate object’s identity. For example, Taborda-Osorio and Cheries [22] demonstrated that 13-month-old infants used internal properties rather than external properties to determine how many agents were involved in an event, but only when the objects had eyes and exhibited self-propelled motion, clear cues to animacy.

In the context of categorization, Welder and Graham [23] demonstrated that the presence of eyes on a novel object directed infants’ attention towards an object’s insides. In this study, 14- to 15-month-old infants were familiarized with novel objects possessing either similar insides or similar outsides in an object examining paradigm. When infants were not given any additional cues regarding category membership, they categorized the items based on their outsides only. However, when eyes were placed on the objects, infants categorized the items based on both internal and external features. Crucially, when a similar perceptual cue was used (a strip of material placed on the objects), infants did not exhibit categorization based on the objects’ insides, suggesting the eyes acted as cues to animacy, rather than as a salient perceptual feature.

In the current study, we asked whether sensitivity to the relevance of object insides for the categorization of animate objects is in place at an earlier age. We tested infants ranging in age from 9 to 11 months, with an average age of 10 months, in light of recent evidence showing that 6.5- to 9.5-month-olds will use animacy cues to guide their expectations of an object’s insides [20]. Our motivation for testing 10-month-olds is guided by Taborda-Osorio and Cheries [19]’s recent proposal that infants’ bias to represent kind membership based on non-obvious properties, as well as to attribute insides to animate beings emerges during the first year of life. As reviewed above, however, little empirical research has directly addressed whether infants under 12 months attend to the insides of animates, with the exception of Setoh et al. [20] who demonstrated that 8-month-olds expect self-propelled objects to have insides. Thus, examining whether 10-month-olds will use shared insides to categorize animate objects could offer critical support for this account and, when considered with other research, offer critical insight into the developmental emergence of attention to insides.

Building on the object examining procedures used by Welder and Graham [23], infants were familiarized with a series of distinct novel objects that could be opened to reveal their internal contents in one of three conditions: with no additional cues (No Cues), an animacy cue condition in which two eyes were placed on top of each object (Eyes), and a perceptual cue control condition in which a small plastic spoon was placed on top of each object (Cue control). The goal of the Cue Control condition was to present infants with a salient perceptual marker that each object possessed that would draw infants’ attention without providing any meaningful information about object kind, allowing us to examine the possibility that any added shared perceptual cue would draw infants’ attention to other shared commonalities (i.e., shared insides). Given that infants of this age would likely have been exposed to the types of eyes used (through stuffed animals for example), we chose a familiar cue to contrast with the eyes. Note that our goal was not to examine whether eyes were superior to other potential animacy cues, such as fur, appendages, other facial features and we thus did not wish to use a cue that is generally fixed to an organism. Rather, we simply wanted to rule out the possibility that any salient shared perceptual feature would provide category information. In addition, we used one item rather than two (e.g., two stickers), as pilot work by Welder and Graham [23] suggested that two pieces of material could be construed as eyes.

Following familiarization, infants were presented with a two novel test objects: an in-category object that shared the same insides as the familiarization objects, followed by the out-of-category test object which shared no properties with the familiarization objects. Using this paradigm, we expected that infants would show evidence of categorization by recovering their examining time significantly more to the out-of-category object than to the in-category object [24, 25].

Our specific predictions were as follows: first, if 10-month-olds, like 14-month-olds, see insides as relevant to animate categories, only infants in the Eyes condition will spend more time examining the out-of-category object than the in-category object, as they see the in-category object as belonging to the same category as the familiarization objects. If, however, infants can readily form a category of novel objects based on shared insides, then both infants in the No Eyes and the Eyes conditions will show similar patterns of object examining. Finally, if any shared cue signals that the objects are similar, then adding either eyes or the spoon should lead infants to categorize the objects together and examine the in-category object less than the out-of-category object.

## Methods

### Participants

Data from 58 infants (*M* = 10.15 months, *SD* = .60, *range* = 9.00 to 10.98 months) were included in the final sample. Participants were randomly assigned to one of three groups: No Cues (n = 20; *M* = 10.16 months, *SD* = .64, *range* = 9.00 to 10.98 months, 11 girls), Eyes (n = 18, *M* = 9.89 months, *SD* = .64, *range* = 9.13 to 10.92 months, 7 girls), and Cue control (n = 20, *M* = 10.35 months, *SD* = .43, *range* = 9.44 to 10.95 months, 10 girls). Sixteen additional infants were tested but excluded from the sample due to experimenter error/failure to complete experiment (*n* = 9; note the data from these infants were not coded) or because they had examining times of zero on the in-category or out-of-category objects (*n* = 7). Infants were from primarily English-speaking homes that varied within the more general middle class (although SES was not formally assessed). Written informed consent was provided by parents. This experiment was approved by the Conjoint Faculties Research Board at the University of Calgary (Project title: Reasoning about object categories and properties during early childhood REB16-0423).

### Stimuli

**Fig 1:**
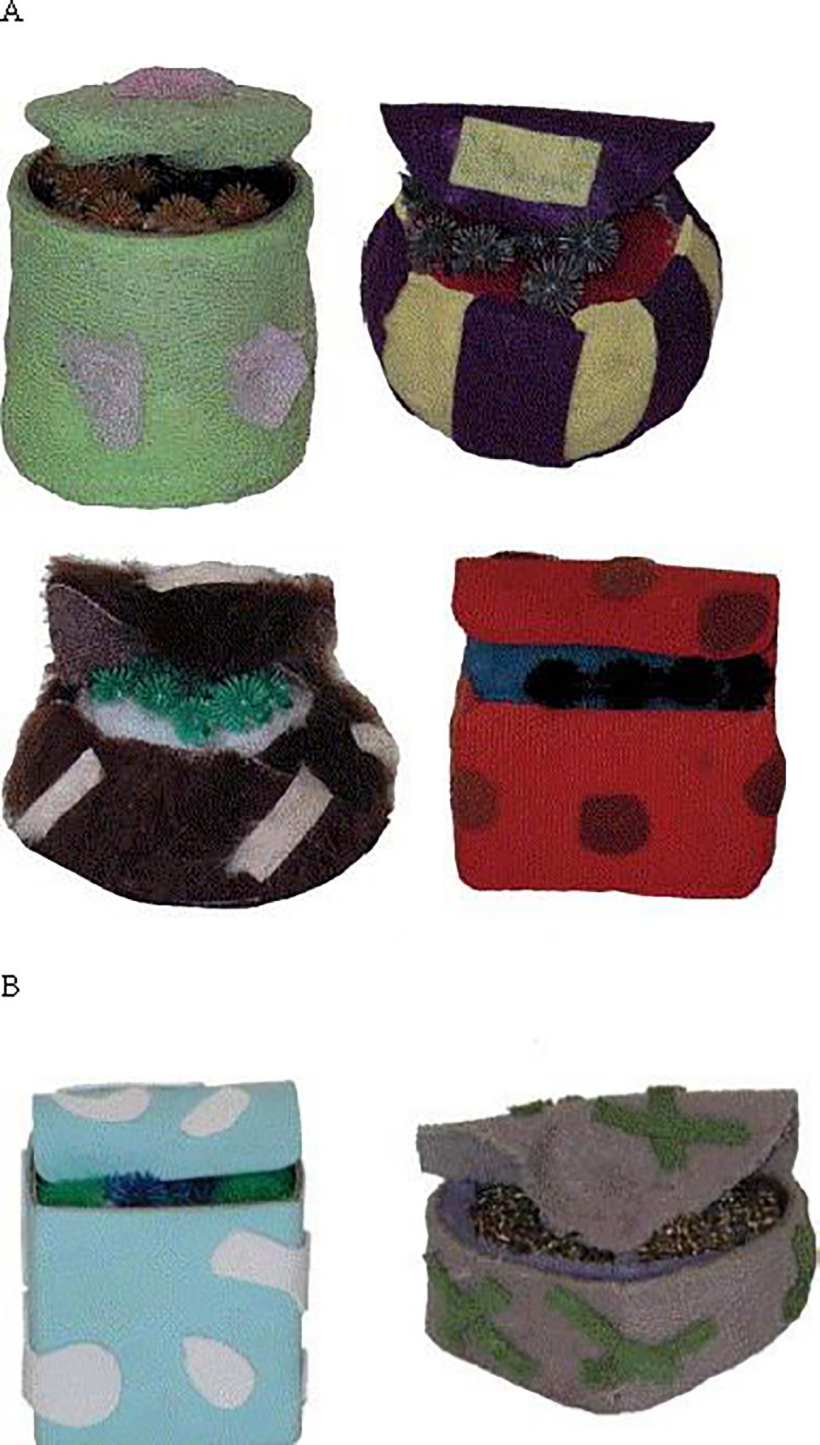
Object set used in the No Cues condition. (A) Familiarization objects. (B) In-category and out-of-category test objects.

**Fig 2:**
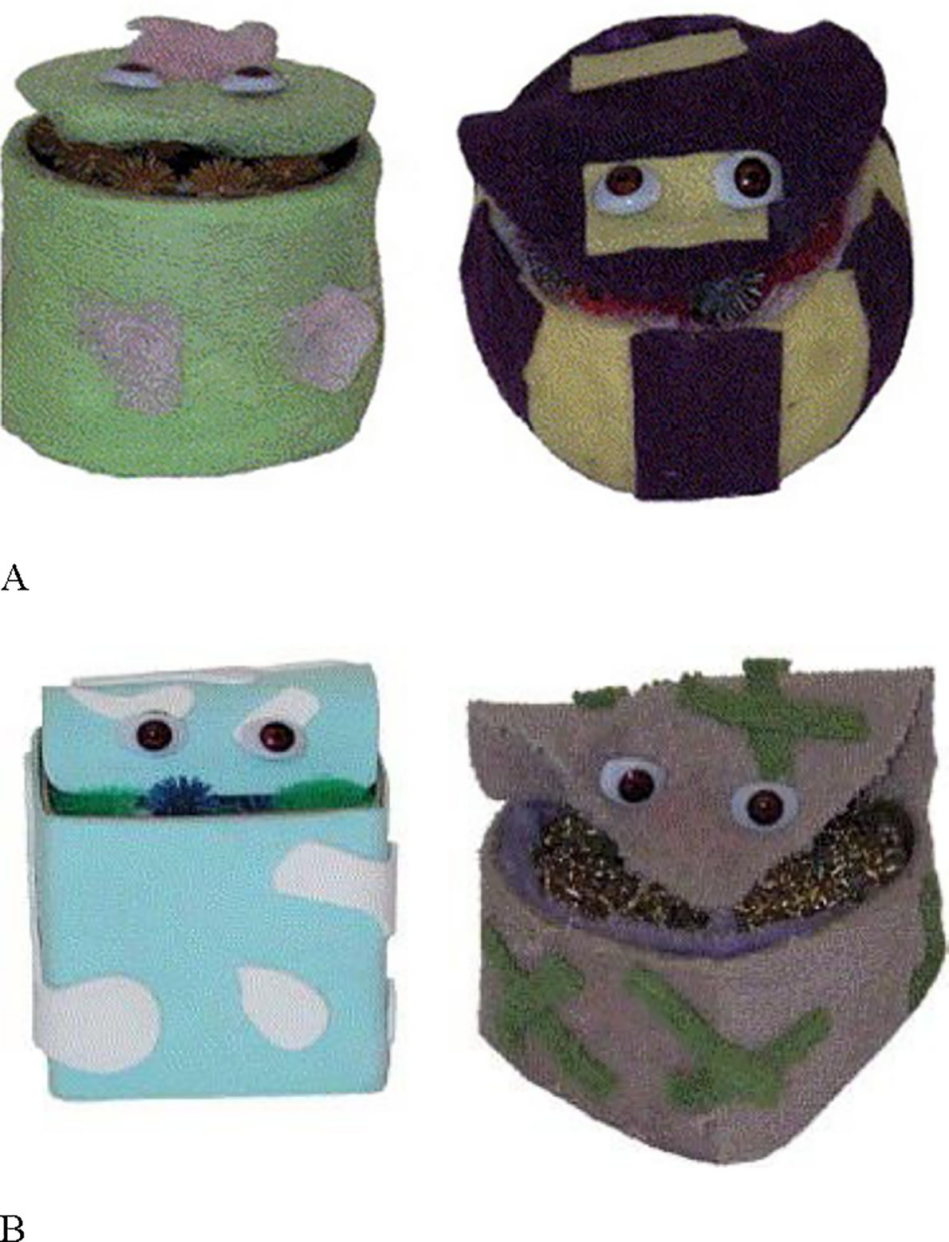
Objects used in the Eyes condition. (A) Example familiarization objects. (B) In-category and out-of-category test objects. Note that the full set of familiarization objects are identical to those used in the No Cues condition, with the exception of the added eyes.

**Fig 3:**
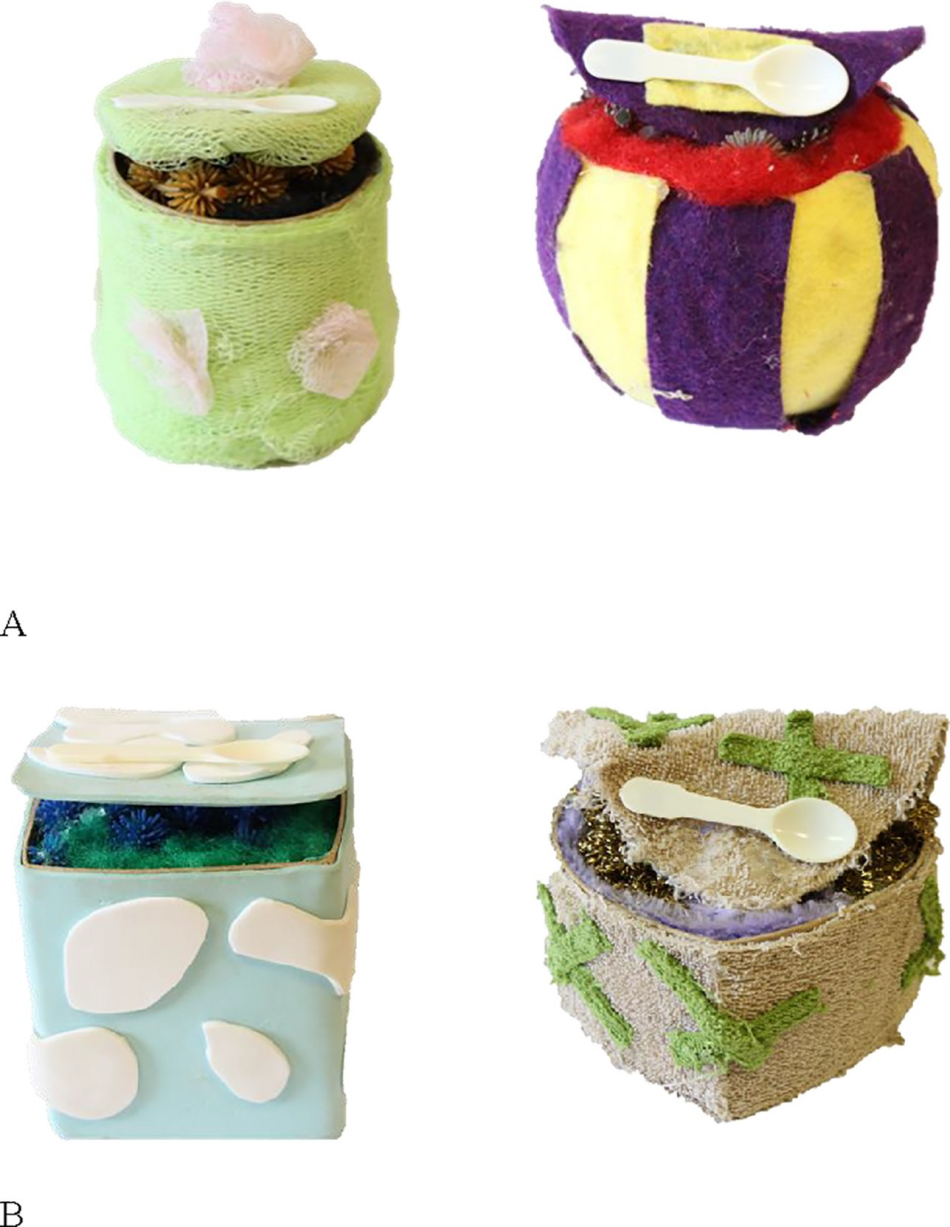
Objects used in the Cue control condition. (A) Example familiarization objects. (B) In-category and out-of-category test objects. Note that the full set of familiarization objects are identical to those used in the No Cues condition, with the exception of the added spoon.

Three sets of objects were used in this experiment: one without any added cues, one with eyes attached, and one with spoons attached (see Figs 1–3 for the object sets). With the exception of the presence or absence of eyes/spoons, the sets were identical. Each set was comprised of six novel objects: four familiarization objects, an in-category test object, and an out-of-category test object. Each object had a top flap that could be opened to expose the insides of the object. The insides of the familiarization objects and the in-category test object were similar, and were created using polyester fluff and connecting rubber spheres with proptruding extensions. The insides in the out-of-category object differed from that of the familiarization objects and in-category object and were creatd using gold scouring pads and mauve pipe clearners. All the objects were discriminable from one another as the outsides differed in shape (rectangle, sphere, cylinder, circle, cube, and heart), size (from 10–13 cm in length, 8–13 cm in width, and 7–12 cm in height), texture (corduroy, felt, mesh, faux fur, foam, and terrycloth), and colour (green and purple, purple and yellow, brown and white, red and burgundy, blue and white, and grey and green). For the object set with eyes on them, all the objects had two realistic-looking plastic doll eyes placed on the top part of the flap. For the object set with spoons, a small plastic white spoon was positioned in the same location as the eyes were placed in the Eyes set.

### Design

The same procedure was used for all three groups with one exception: the object set presented. The object examining task began with a familiarization phase (8 trials) followed by a test phase (2 trials), for a total of 10 trials, each 30 seconds in duration. Pilot research indicated that 30 seconds allowed sufficient time for infants to explore the objects and become familiarized. Furthermore, using fixed intervals of 30 seconds allowed for direct comparisons of examining time during familiarization and during the test trials.

The familiarization phase consisted of two blocks of four trials each. In each block, the four familiarization objects were presented, one on each trial. The order in which the four objects were presented was counterbalanced across infants. The two test trials were presented in a fixed order: the in-category object test trial, the out-of-category test trial. This order was held constant to ensure that any increase in examining time to the out-of-category object could be attributed to category formation. That is, if the out-of-category object had been presented first, infants may have increased their examining time to this object simply because it was novel and not because they viewed it as a member of a new category (see [26, 27] for a discussion).

### Procedure

Infants were seated on their parent’s lap at a table across from the experimenter. Parents were instructed to speak to their infants as little as possible, and not to point out or show the objects to their infant. Parents were also instructed to silently place objects within their infants’ reach, if infants handed the objects to the parents or dropped the objects on the floor.

Each object was presented one at a time for 10 trials, for 30 seconds each. The experimenter used a small stopwatch to time these intervals. The procedure for each trial was identical: each time an object was presented the experimenter said, “Hi (infant’s name)! See this! Look here at this! Look at this!” while waving her hand over the outside of the object. This dialogue was repeated as the experimenter lifted the flap of the object and waved her hand over the inside of the object. The presentation order of the insides and outsides of the object was counterbalanced across trials and infants. Once the introduction was completed, the experimenter placed the object on the table, within the infant’s reach. Infants were allowed to examine objects for 30 seconds. Objects were designed such that the flap remained slightly open and the contents of the insides were visible. In the event that the infant dropped the object or passed the object to the parent or the experimenter, the object was retrieved and placed back within the infant’s reach. We did not compensate for time lost due to these actions, as they are considered to reflect the infants’ intentional actions of disinterest [28]. After 30 seconds, the experimenter removed the object and proceeded to the next trial. Testing sessions were recorded for coding purposes, using a video camera hidden behind a one-way mirror.

### Coding and Data Reduction

A coder, unaware of the hypotheses of the experiment, recorded object examining time for each of the 10 trials, using the video recordings. Object examining time was defined as the duration of time spent actively looking at and/or physically manipulating an object (see [24], [29]), thus incorporating both looking at the object and manipulating the object. See S1 Appendix for a detailed description of behaviours coded. The time an infant spent examining an object was totaled to provide an object examining score for each object.

To calculate inter-rater reliability, 24% of the data (*n* = 14) was recoded by a second coder, also unaware of the experimental hypotheses. Interrater reliability was high, *ICC* = . 97, *p* < .0001.

## Results

### Familiarization trials

In the first set of analyses, object examining times during the first and second blocks of the familiarization phase were first compared to assess whether infants significantly decreased their object examining across the familiarization trials. Results of a 3 (Group: No Eyes, Eyes, and Cue control) x 2 (Block 1, Block 2) mixed factor ANOVA yielded a main effect of group, *F*(2, 55) = 4.598, *η*_*p*_^2^ = .143, *p* = .014. Follow-up analyses using LSD comparisons indicated that infants in the Cue control group (*M* = 16.67, *SD* = 3.87) had longer examining times, collapsed across the two blocks, than infants in the No Eyes group (*M* = 12.70, *SD* = 3.98), *p* = .004. The Eyes group (*M* = 14.29, *SD* = 4.64) did not differ significantly from the Cue Control (*p* = .09) nor the No Eyes group (*p* = .24). Most relevant for our research question, the ANOVA yielded a significant main effect of block, *F*(1, 55) = 52.508, *η*_*p*_^2^ = .488, *p* < .0001. As expected, infants in all three groups decreased their examining times from Block 1 to Block 2, thus demonstrating that they became familiarized to the objects. The group by block interaction was not significant *F*(2, 55) = .1.11, *η*_*p*_^2^ = .04, *p* = .337.

Next, we examined the decline in object examining from Trial 1 (the first familiarization trial) to Trial 8 (the last familiarization trial) to examine whether there were group differences in how quickly infants became familiarized. A one-way ANOVA using these difference scores indicated that there were no group differences in rate of decline in object examining, *F*(2, 55) = .023, *η*_*p*_^2^ = .001, *p* = .977. Thus, infants’ rate of familiarization did not vary according to the object set presented.

### Test trials

**Fig 4:**
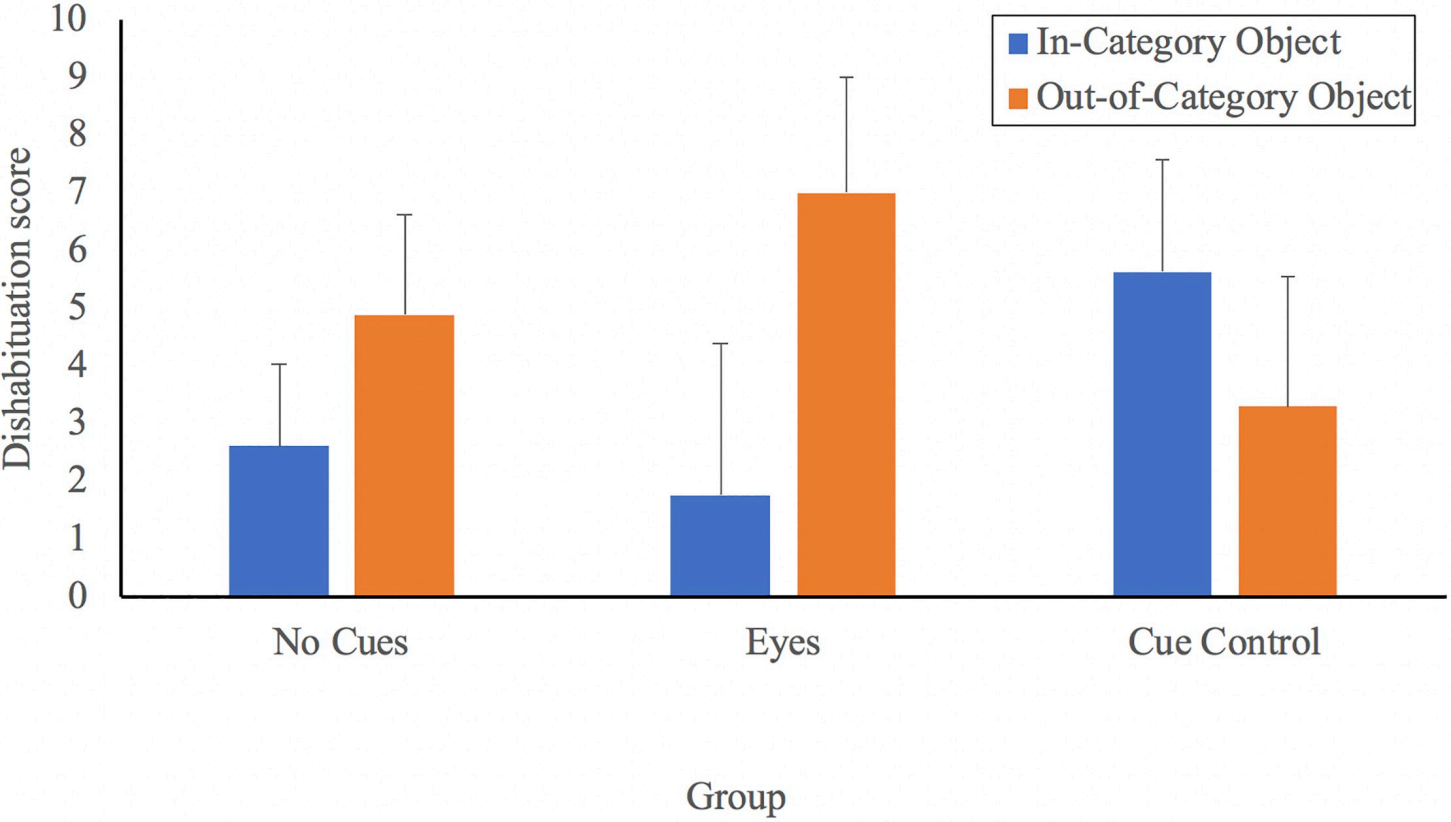
Dishabituation scores as a function of test object and group.

To examine infants’ category formation, dishabituation scores were used (see [24]). The in-category score was calculated by subtracting infants' object examining time for the final familiarization trial from that for the in-category test trial. Similarly, the out-of-category score was calculated by subtracting examining time for the last familiarization trial from that for the out-of-category test trial. We used these dishabituation scores in our analyses as they are considered a more accurate reflection of category discrimination than raw examining times. That is, dishabituation scores reflect relative changes in each infant's examining times, taking into account individual differences in interest, exploration, and attention [24, 30]. Dishabituation scores for the three groups are presented in Fig 4.

A 3 (Group) by 2 (Score: In-category vs. Out-of-Category) mixed factors ANOVA indicated no significant main effects of group, *F*(2, 55) = .048, *η*_*p*_^2^ = .002, *p* = .953, nor score, *F*(1, 55) = 2.478, *η*_*p*_^2^ = .043, *p* = . 121, but did indicate a significant group by score interaction, *F*(2, 55) = 4.006, *η*_*p*_^2^ = .127, *p* = .024. In line with our predictions, we used paired t-tests comparing recovery to the in-category object versus recovery to the out-of-category object, separately for each group, with a Bonferroni correction (alpha = 0.0167). Infants in the Eyes group displayed significantly more recovery from the last familiarization object to the out-of-category object (*M* = 7.00, *SD* = 8.56) than to the in-category object (*M* = 1.77, *SD* = 11.19), *t* (17) = 2.677, *d* = .516, *p* = .016. Consistent with these results, a binomial test indicated that the majority of infants (14/18) in the Eyes group demonstrated more recovery to the out-of-category object than to the in-category object than would be expected by chance, *p* = .031, two-tailed. In contrast, infants in the No Eyes group did not exhibit any significant differences in object examining recovery to the in-category object (*M* = 2.62, *SD* = 6.42) versus the out-of-category object (*M* = 4.89, *SD* = 7.79), *t* (19) = 1.239, *d* = .315, *p* = .231. Similarly, infants in the Cue Control group did not exhibit any significant differences in object examining recovery to the in-category object (*M* = 5.64, *SD* = 8.69) versus the out-of-category object (*M* = 3.30, *SD* = 10.11), *t* (19) = 1.230, *d* = .246, *p* = .234. Binomial tests indicated that the number of infants in the No Eyes group (11/20) and the Cue control group (9/20) that demonstrated more recovery to the out-of-category object than to the in-category object did not differ from that expected by chance, *p =* .824, and *p =* .824, both two-tailed. Taken together, these results suggest that only infants in the Eyes group discriminated the in-category object from the out-of-category object.

## Discussion

Here we examined whether an animacy marker would facilitate 10-month-old infants’ categorization of novel objects with shared insides. Infants were familiarized with novel objects that had different outward appearances, but shared similar internal features. Test trials involved presenting infants with a novel in-category test object, followed by a novel out-of-category test object. Category formation was assessed using dishabituation scores, whereby infants were considered to have formed a category for the objects if they exhibited significantly greater recovery to the out-of-category test object than to the in-category object. Results indicated that infants only categorized the objects when they were presented with an animacy cue, namely, eyes placed on the top of the objects. Critically, infants in the Cue control (i.e., spoons) group did not categorize the items, suggesting that it was not merely the presence of a shared surface feature that promoted infants’ categorization of the novel objects with less obvious features.

Together, the results indicate that when objects are presented with animacy cues, 10-month-olds infants will group together novel objects that share inside features. Infants’ attention to inside features for objects bearing animacy cues is consistent with research with preschoolers demonstrating the importance of internal features for categorization of animate kinds (e.g., [12, 13, 16, 17]). Critically, these findings add to a growing body of research demonstrating that the expectation that insides are relevant for animate objects emerges during the first year of life [20–23].

This developmental continuity from infancy to preschool, however, does not imply that infants share the same causal expectations about insides features that preschoolers do. Instead, we favor the account recently put forth by Taborda-Osorio and Cheries [19] which proposes that that there are developmental precursors to later biological understanding which are present in infancy. That is, Taborda-Osorio and Cheries [19] argue that older children’s tendency to focus on internal features when reasoning about animate entities is rooted in two cognitive processes that emerge in the first year of life: a domain-general bias to represent kind membership based on non-obvious properties, and a domain-specific tendency to attribute physical insides to animate entities. Our findings support this proposition by demonstrating that young infants will use animacy cues to guide their attention towards the insides of an object within the first year of life, supporting the notion that a more sophisticated understanding of biological kinds may have its roots in certain cognitive biases present in early infancy.

These early tendencies may serve to support the development of later knowledge and reasoning about animate entities. Continuing to study these biases in younger age groups is critical, as this line of inquiry offers insight into the emergence of naïve theories of biology and biological essentialism.

Our results also demonstrate that eyes function as a crucial animacy cue for young infants, consistent with the results of Welder and Graham [23] with 14- to 15-month-old infants. Previous studies have shown that self-propelled motion and agency are markers of animacy for infants (e.g., [20, 21]). In addition, Taborda-Osorio and Cheries [22] demonstrated that eyes would function as an animacy cue when paired with self-propelled motion, but left open the question of whether eyes would function as an animacy cue on their own. The findings from the current research suggest that eyes *can* function as an animacy cue in their own right, as they direct infants’ attention towards the insides of an object.

The current findings contrast with the findings from Setoh et al. [20] indicating that 8-month-olds only expect an object to have insides when it exhibits self-propelled motion and has an physical animacy cue (i.e., fur). That is, Setoh et al. [20] found that an animacy cue on the surface of an object was not sufficient to direct infants’ attention towards the object’s insides when it was presented on its own. There are, however, two important differences between this study and the current research. First, Setoh et al. [20] examined infants that were two months younger than the infants in the present study. Perhaps during these two months of development infants’ cognitive capacity improves in a manner that enables them to better understand and incorporate animacy cues. Second, Setoh et al. [20] used fur as a surface animacy cue, while the current study used eyes. It could be that eyes are considered more indicative of animacy than fur. This would make sense given that various inanimate objects can have furry exteriors (e.g., a fluffy rug), while it is less common for animate objects to have eyes unless they are a representation of an animate being (e.g., a teddy bear). To our knowledge, the only research that has examined eyes and fur separately as animacy cues was conducted with 3-month-old Japanese macaques. Here, fur alone was used an animacy cue by the infant monkeys, while eyes alone were not [31]. However, the authors suggest that the recognition of fur as an animacy cue may simply precede that of eyes in very young infants, as fur constitutes a more global and noticeable feature, while eyes are more distinctive and localized. In addition, eyes represent a crucial social cue for human infants, such that early eye scanning predicts concurrent and later social skills [32, 33]. Adults also consider eyes to be more important than other facial features when determining whether a face appears to be alive [34]. These findings suggest that fur/texture may be a more important animacy cue for infant animals and perhaps very young human infants, while eyes may increase in importance for animacy determination across development. However, just as future research needs to examine eyes and self-propelled motion seperately as animacy cues [22], the relative importance of eyes and fur as animacy cues for human infants remains an empirical question. Other potential cues such as appendages and other facial features should also be explored in future work, in order to fully conceptualize how infants use various features to determine animacy.

Although our findings indicate that 10-month-olds will categorize objects with animacy cues based on their shared insides, this does not suggest that infants will disregard the outside appearance when categorizing objects or animals. Indeed, a large of body of research demonstrates that shared external features guide young infants’ cateogorical decisions (e.g., [35–37], see [38] for a review). For example, infants will catgorize cats and dogs based on the head region [39,40]. Instead, our findings and those of others [20, 22] indicate that once animacy cues are provided, infants will expand the characteristics that they consider when categorizing. It remains unclear, however, whether infants, like preschoolers would priviledge inside information over external properties when categorizing animals [13–15]. However, Taborda-Osorio and Cheries’s [22] findings showing that internal features are given priority over external features when individuating agents could suggest that infants may also privilege internal over external information when categorizing animate beings.

Discussion of our findings also warrants consideration of the potential limitations of using spoons as the perceptual control. Recall that the goal of the Cue control condition was to present infants with a salient perceptual marker that would draw their attention without providing any meaningful information about object kind. Therefore, the control condition was used to allow us to rule out the possibility that any added shared perceptual cue would lead infants to attend to other object commonalities (i.e., insides). As infants have had experience with eyes, we selected a familiar item (spoons) as a contrast condition. It is possible, however, that infants’ familiarity with spoons may have led them to recognize that spoons are typically not attached to or part of other objects, which may have led infants to direct more attention to the objects. If infants were surprised that spoons were attached to the objects, this would likely have resulted in more examining time to the objects during familiarization, relative to the other condition that had an added cue, namely the Eyes condition. Our results do not support the notion that infants were confused by the presence of spoons, as examining time did not differ significantly between the Cue control and Eyes group in the familiarization trials, suggesting that both conditions seemed to have drawn children’s attention similarly. Even if infants did spend more time examining the objects with spoons during familiarization (and we note that overall examination time was significantly longer in the Cue control condition than in the No Eyes conditions during familiarization and was numerically greater than in the Eyes condition (*p* = .09) ), this examination did not lead them to group the objects together based on similar insides. Future research examining the influence of different types of added cues, including biological markers (e.g., facial features, appendages) and unfamiliar cues (e.g., stickers) on infants’ categorization will offer further insight into infants’ expectations about shared insides. Furthermore, comparing the effect of other perceptually salient cues, including those that would be perceived as more integral to the objects than spoons (e.g., handles) to that of animacy cues will offer further insight into the range of cues that may orient infants to the shared inside features of objects.

In conclusion, the findings from this study suggest that 10-month-old infants use eyes as a cue to group together objects based on less obvious similarities. That is, by 10 months of age, eyes signal to infants that objects share some kind of uniting commonality that may not be obvious or readily perceptually available.

## Supporting information

S1 Appendix Coding scheme for object examining. (PDF)
