# Supplementary material for: Animacy cues facilitate 10-month-olds' categorization of novel objects with similar insides
Source: PLoS One. 2018 Nov 26;13(11):e0207800. doi: 10.1371/journal.pone.0207800 (PMC6261258; doi:10.1371/journal.pone.0207800)
Supplement: S1 Appendix — (PDF) [file pone.0207800.s001.pdf]

S1 Appendix

Coding Scheme for Object Examining

This coding scheme was adapted from from Welder & Graham (2004). Both active looking and manipulation were coded. *Active looking* was only coded when looking at the object was accompanied by a facial expression that denoted concentration (i.e., a furrowed brow, a focused gaze, or facial movements such as pursed lips). Object examining was not coded if the infant did not appear to be looking at the object with interest or was looking at the object while engaged in showing it to the experimenter or parent, or throwing it (see Oakes et al., 1991). *Manipulation* was coded when the infant made physical contact with the object, including picking up, shaking, rotating, stroking, squeezing, poking, gripping, or moving the object from hand to hand. The infant did not have look directly at the object while manipulating it for object examining to be coded, but the manipulation had to be purposive. For example, object examining was not coded if the infant looked around the room with a hand resting on the object or if the infant was banging the object on the table or opening and closing the flap repeatedly. Finally, object examining did not include mouthing or rubbing the object on the hair or face, as these behaviors do not reflect attention to objects in infancy (see Ruff, 1984, 1986).
